# The first case of thrombocytopenia, anasarca, fever, renal impairment or reticulin fibrosis, and organomegaly (TAFRO) syndrome with unilateral adrenal necrosis: a case report

**DOI:** 10.1186/s13256-018-1814-9

**Published:** 2018-10-08

**Authors:** Yu Fujiwara, Kanae Ito, Akito Takamura, Kaoru Nagata

**Affiliations:** 1 Department of Internal Medicine, Musashino Red Cross Hospital, 1-26-1, Kyonancho, Musashino-shi, Tokyo, 1808610 Japan; 2 Department of Rheumatology and Collagen Disease, Musashino Red Cross Hospital, Tokyo, Japan; 3 Department of General Internal Medicine, Musashino Red Cross Hospital, Tokyo, Japan

**Keywords:** TAFRO syndrome, Castleman disease, Adrenal necrosis, Tocilizumab

## Abstract

**Background:**

TAFRO syndrome, which was first reported in 2010 in Japan, is a relatively rare disease characterized by thrombocytopenia, anasarca, fever, renal impairment, reticulin fibrosis, and organomegaly. Although this disease is considered similar to multicentric Castleman disease, some of the clinical features, such as thrombocytopenia, are different from typical cases of multicentric Castleman disease. In addition, the etiology of TAFRO syndrome remains unknown and controversial. There have only been a few cases of TAFRO syndrome complicated with adrenal gland lesions, and all of them have had hemorrhagic involvement.

**Case presentation:**

This report describes the case of a 46-year-old Asian man who presented with fever, epigastric pain, and back pain for 1 month. A computed tomographic scan revealed ascites, mild lymphadenopathy, and left adrenal necrosis without hemorrhage. A blood test showed thrombocytopenia, anemia, and elevated C-reactive protein, alkaline phosphatase, and creatinine levels. Based on the edema, severe thrombocytopenia, fever, reticulin myelofibrosis shown by bone marrow biopsy, mild lymphadenopathy, and progressive renal insufficiency, we diagnosed this patient as having TAFRO syndrome. He was successfully treated by immediate administration of glucocorticoids and tocilizumab.

**Conclusions:**

There have been no previous reports of a case of TAFRO syndrome complicated with adrenal necrosis. Because the biopsy of the left adrenal gland revealed necrosis without any evidence of hemorrhage, we concluded that the unilateral adrenal necrosis in this case was caused by either ischemia from infarction or organomegaly itself under severe hypercytokinemia. This unusual clinical course is useful for further analysis of the etiology of TAFRO syndrome.

## Background

TAFRO syndrome (thrombocytopenia, anasarca, fever, renal impairment or reticulin fibrosis, and organomegaly) was first reported by Takai *et al*. in 2010 [1], and several cases have since been reported, mainly in Japan, but also in other countries. At first, TAFRO syndrome was thought to be a subtype of human herpesvirus 8 (HHV-8)-negative idiopathic multicentric Castleman disease (iMCD), but Iwaki *et al.* recently reported that the pathophysiology of TAFRO syndrome seemed to differ from that of iMCD [2]. Along with increasing case reports, the diagnosis criteria and treatment guidelines, as well as the concept of this disease, have been established gradually [2, 3]. In terms of organ damage, the kidneys are commonly involved, and temporal hemodialysis is required in some cases [2]. Notably, there have only been a few cases of TAFRO syndrome complicated with adrenal lesions, and all of them have had hemorrhagic involvement [4, 5]. No cases accompanied by adrenal gland necrosis without hemorrhage have yet been reported.

This is the first case of TAFRO syndrome complicated with unilateral adrenal necrosis proven pathologically. Our findings reveal the possibility of ischemic involvement with this unusual unilateral adrenal necrosis. Because the etiology of TAFRO syndrome is still unclear and controversial, further analysis is necessary to understand the clinicopathology of this disease, and this case will be beneficial for investigations into the pathophysiology of TAFRO syndrome.

## Case presentation

**Fig. 1:**
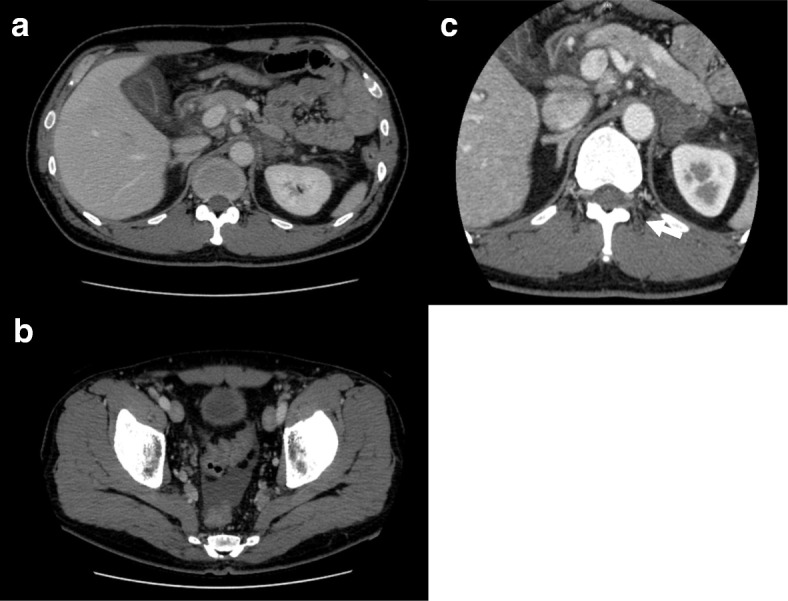
The images of the first contrasted computed tomography scan. **a** Edema around the gallbladder. **b** Ascites. **c** Left adrenal gland without the contrast effect (*arrow*). Organomegaly including hepatosplenomegaly and lymphadenopathy is not seen here

A 46-year-old Asian man without any significant past medical history presented to an out-patient clinic complaining of fever, epigastric pain, and back pain. He was diagnosed as having gastric ulcer by upper gastrointestinal endoscopy and prescribed a proton pomp inhibitor; however, his fever of approximately 38 °C and his back pain remained. Two weeks later, his back pain had worsened, and the laboratory data of the out-patient clinic showed an elevated C-reactive protein level (17.2 mg/dL); thus, he came to our hospital for further evaluation. His medication included only orally administered azelnidipine for hypertension. There was no significant family medical history. He denied smoking tobacco, alcohol consumption, and exposure to toxins. He worked at a ceremonial hall without any ill contacts. He had a fever of 37.9 °C, heart rate of 90 beats per minute (bpm), respiratory rate of 20 breaths/minute, blood pressure of 126/78 mmHg, and oxygen saturation of 97% on room air. A physical examination including a neurological examination showed a well man without any specific abnormal findings. Blood tests at the first encounter revealed a white blood cell count of 10,300/μL with 70% neutrophils, 14% lymphocytes, and 16% monocytes, and the platelet count was 275,000/μL. His lactate dehydrogenase level was 299 IU/L (normal range, 119–229 IU/L), his alkaline phosphatase level was 983 U/L (normal range, 103–335 U/L), and his gamma-glutamyl transpeptidase level was 256 IU/L (normal range, 0–73 IU/L). His C-reactive protein level was 23.47 mg/dL (normal range, 0–0.29 mg/dL). Other results are shown in Table 1. A contrasted computed tomography (CT) scan showed edema around his gallbladder without gallstones or bile duct dilation, along with left adrenal enlargement without contrast, suggesting necrosis and slight pleural effusion (Fig. 1). His right adrenal gland was contrasted normally. Contrasted magnetic resonance imaging (MRI) of his adrenal glands was also performed, and the results showed necrosis of his left adrenal gland with a slight possibility of infarction and no specific evidence of hemorrhage. He was hospitalized for further investigation into the cause of the unilateral adrenal necrosis.

**Table 1:** Laboratory data at first hospitalization and rehospitalization

Hospitalization	1st	2nd	
Complete blood cell			
WBC	10300	11200	/μL
Neutro	70.0	71.0	%
Lympho	14.0	16.0	%
Mono	16.0	13.0	%
RBC	531	517	10^4^/μL
Hgb	14.7	14.0	g/dL
PLT	275000	5000	/μL
Immunochemistry			
CRP	23.5	31.9	mg/dL
Anti-SS-A Ab		113	U/mL
Anti-dsDNA Ab		Negative	
ANA Speckled	1:80		
Cytoplasm	1:40		
PR3-ANCA		< 1.0	
MPO-ANCA		< 1.0	
PAIgG		1310	ng/10^7^cell
IgG	1514	1497	mg/dL
IgA	226.1	194.6	mg/dL
IgM	115.9	79.6	mg/dL
IgG4		73.6	mg/dL
C3	170.9	133.2	mg/dL
C4	35.7	25.7	mg/dL
CH50	47.0	47.2	mg/dL
ADAMTS-13		61	%
AMA M2	19		
Biochemistry			
TP	6.8	6.0	g/dL
Alb	2.7	2.1	g/dL
LDH	299	347	IU/L
T-Bil	0.3	0.6	mg/dL
AST	26	36	U/L
ALT	25	37	U/L
ALP	983	1410	U/L
γGTP	256	247	U/L
BUN	10.5	16.3	mg/dL
Cr	0.85	1.03	mg/dL
Na	140	140	mEq/L
K	4.5	4.1	mEq/L
Cl	106	107	mEq/L
eGFR	77.2	62.6	mL/min/L
Coagulation test			
PT-INR	1.19	1.26	
APTT	43.0	47.6	second
Fibrinogen	> 600	> 600	mg/dL
FDP		28.3	μg/mL
D-Dimer	5.1	8.3	μg/mL
AT-III	63	69	%
Anti-β2GPI Ab	< 1.2	< 1.2	
LAC (dRVVT)	1.16	1.33	
Coombs test		Negative	

*Ab* antibody, *ADAMTS-13* a disintegrin-like and metalloproteinase with thrombospondin type 1 motifs 13, *Alb* albumin, *ALP* alkaline phosphatase, *ALT* alanine aminotransferase, *AMA* antimitochondrial antibody, *ANA* antinuclear antibody, *APTT* activated partial thromboplastin time, *AST* aspartate aminotransferase, *AT-III*antithrombin III, *BUN* blood urea nitrogen, *CH50* 50% complement hemolytic unit, *Cr* creatinine, *CRP* C-reactive protein, *dRVVT* diluted Russell’s viper venom time, *ds-DNA* double-stranded DNA, *eGFR* estimated glomerular filtration rate, *FDP* fibrinogen degradation products, *Hgb* hemoglobin, *LAC* lupus anticoagulant, *LDH* lactate dehydrogenase, *Lympho* lymphocytes, *Mono* monocytes, *MPO-ANCA* myeloperoxidase-ANCA, *Neutro* neutrocyte, *PAIgG* platelet-associated immunoglobulin G, *PLT* platelet, *PR-3 ANCA* proteinase-3 anti-neutrophil cytoplasmic antibody, *PT-INR* prothrombin time-international ratio, *RBC* red blood cell, *SS-A* Sjögren syndrome-A, *T-Bil* total bilirubin, *TP* total protein, *WBC* white blood cell, *β2GPI* beta-2-glycoprotein I, *γGTP* γ-glutamyl transpeptidase

**Fig. 2:**
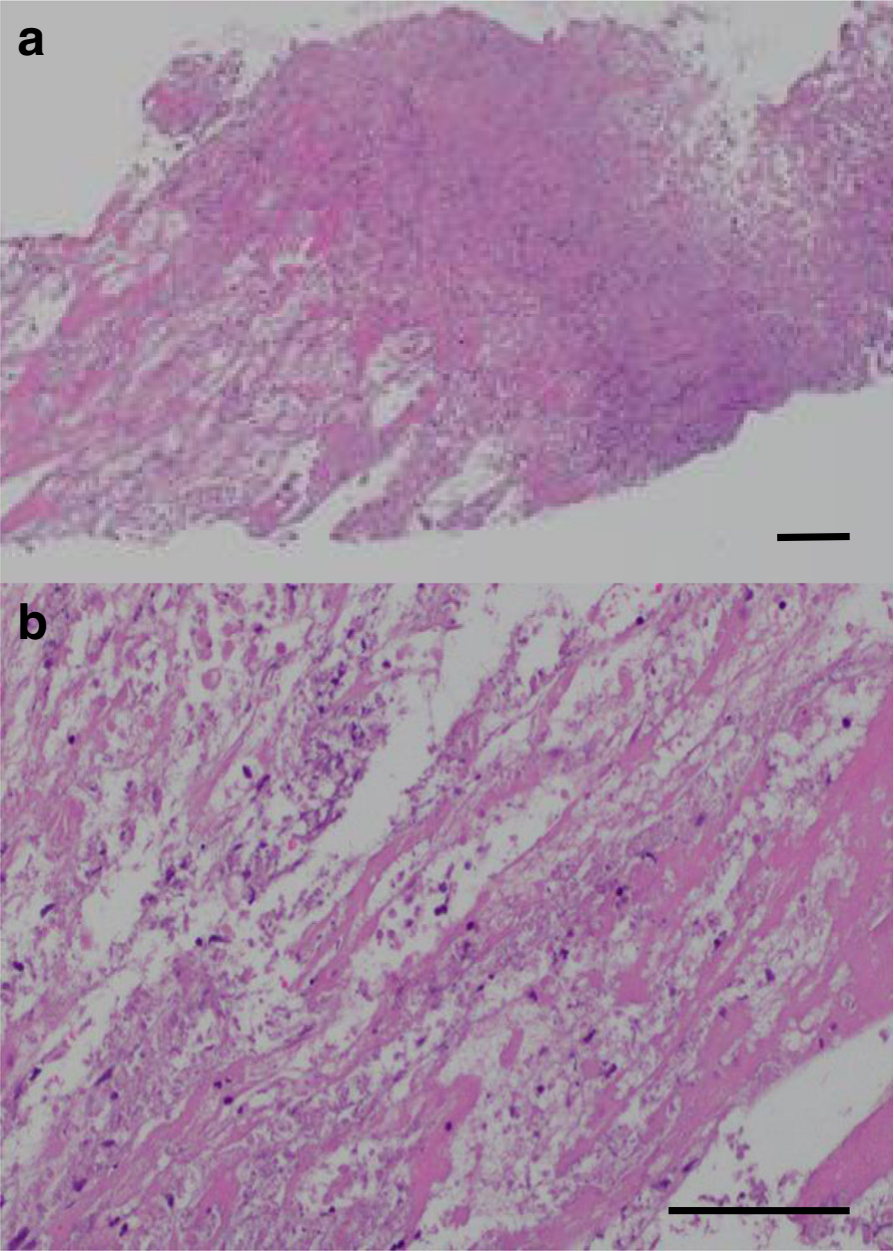
**a**, **b** Histological findings of the left adrenal gland by computed tomography-guided needle biopsy. Necrosis of the left adrenal gland is seen. There is no hemosiderin accumulation, suggesting a low possibility of a hemorrhagic etiology. (Hematoxylin and eosin staining; scale bar, 100 μm)

**Fig. 3:**
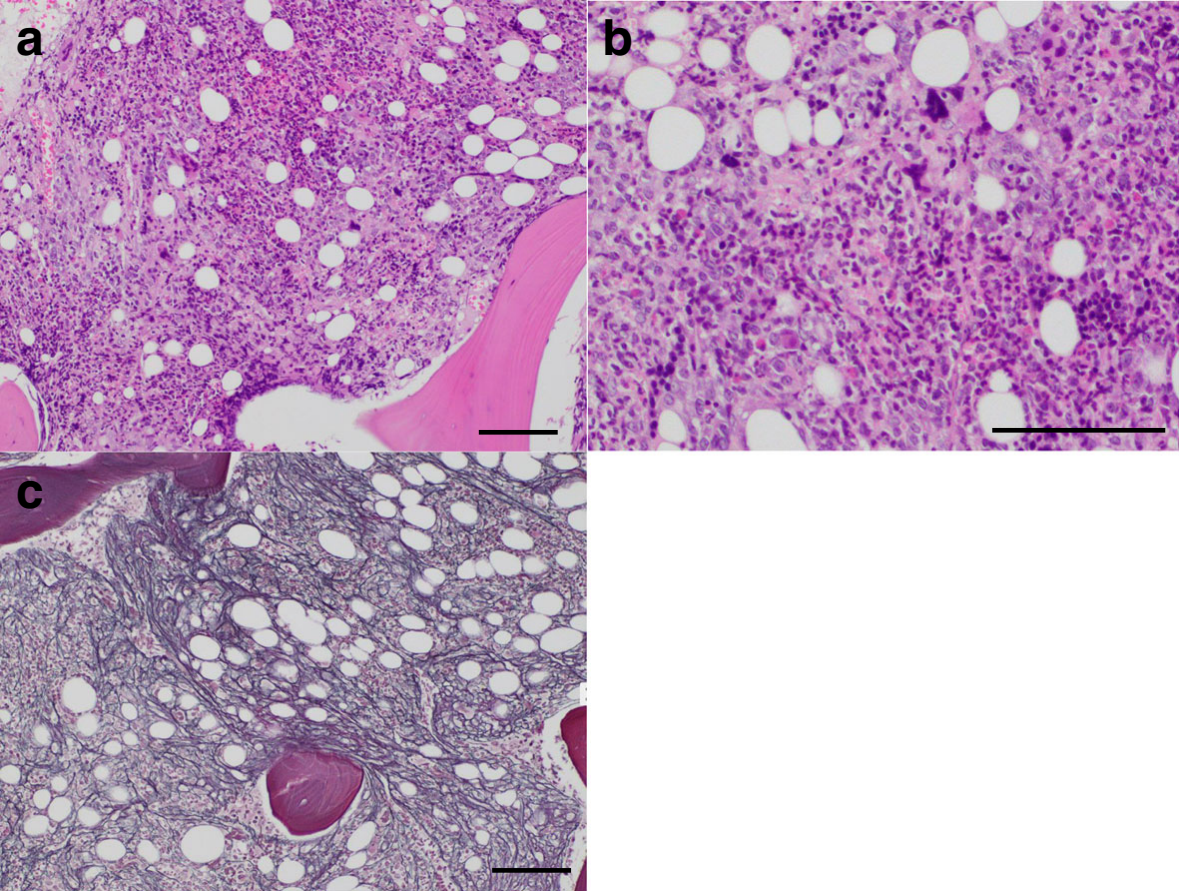
Histological findings for bone marrow. **a**, **b** Hypercellular marrow with fibrosis and an increase in megakaryocytes are seen. (Hematoxylin and eosin staining.) (**c**) Increased and crossing of reticulin fibers form the MF-1 fibrosis. (Silver staining; scale bar, 100 μm)

**Fig. 4:**
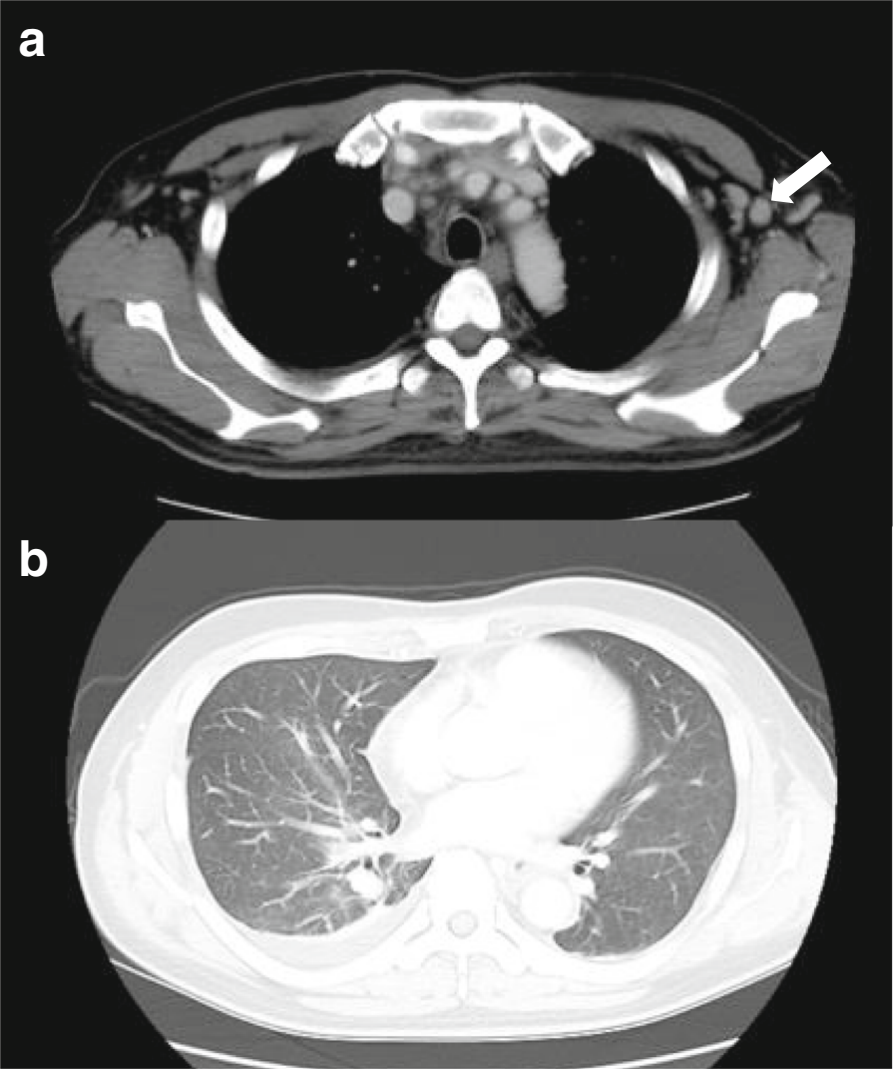
Images of the second contrasted computed tomography scan. **a** Left axillary lymphadenopathy (15 mm) (*arrow*). **b** Pleural effusion

**Fig. 5:**
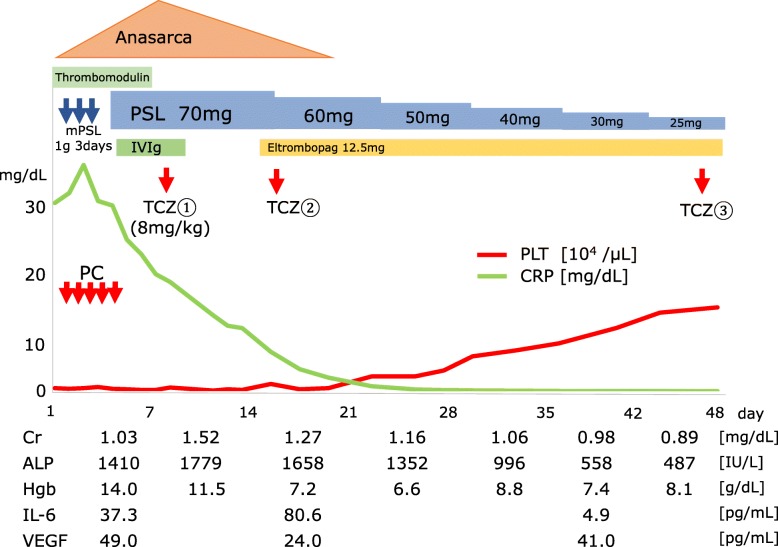
Clinical course. *ALP* alkaline phosphatase, *Cr* creatinine, *CRP* C-reactive protein, *Hgb* hemoglobin, *IVIg* intravenous immunoglobulin, *IL-6* interleukin-6, *mPSL* methylprednisolone, *PC* platelet concentrate, *PLT* platelet, *PSL* prednisolone, *TCZ* tocilizumab *VEGF* vascular endothelial growth factor

Lupus anticoagulant and don't break the value complex antibody were measured, and both were negative, which suggested a low possibility of antiphospholipid syndrome. He did not meet the criteria for diagnosis of systemic lupus erythematosus. We considered the possibility of adrenal insufficiency or pheochromocytoma and measured several types of adrenal hormones, such as serum cortisol, adrenocorticotropic hormone, plasma renin activity, plasma aldosterone activity, and urinary metanephrine and normetanephrine, but none of them explained our patient’s condition. The culture results from blood drawn at the first encounter were all negative. We performed CT-guided needle biopsy of his left adrenal gland, which revealed necrosis and the formation of fibrotic granulomatous tissue (Fig. 2). There was no epithelioid granuloma, malignant lymphoma cells, or hemosiderin deposition, suggesting a low possibility of the involvement of a hemorrhagic etiology. The bacterial culture of this biopsy tissue was also negative. After the biopsy was finished, he was discharged. However, 1 week later, severe thrombocytopenia (5000/μL) appeared, and he was rehospitalized. His creatinine level had increased to 1.03 mg/dL from the initial value of 0.85 mg/dL. Bone marrow aspiration first resulted in a dry tap, but subsequent results showed increased megakaryocytes and hypercellular marrow with fibrosis classified as MF-1 according to the European consensus on bone marrow fibrosis staging (Fig. 3). A contrasted CT scan showed new left axillary lymphadenopathy with a size of 15 mm, right pleural effusion, and increased ascites (Fig. 4). Because our patient’s condition was worsening, we needed to start immediate treatment for any possible underlying causes, including bacterial infection and autoimmune disease, before obtaining the exact diagnosis. The laboratory data from the second hospitalization are shown in Table 1. The clinical course of this case is shown in Fig. 5. The initial treatment included ampicillin/sulbactam and a methylprednisolone pulse followed by orally administered prednisolone and intravenous immunoglobulin therapy (400 mg/kg for 5 days), considering the underlying causes mentioned above, such as severe bacterial infection or autoimmune diseases including antiphospholipid syndrome and immune thrombocytopenia; however, all of these treatments seemed to be ineffective. We also used recombinant thrombomodulin (380 U/kg) for 7 days to cope with the possibility of a thrombotic event or disseminated intravascular coagulation. Because the blood and adrenal gland biopsy culture results were both negative, we stopped the antibiotic treatment. On hospital day 9, we performed a left axillary lymph node needle biopsy, which showed no evidence of malignant lymphoma. With the edema, severe thrombocytopenia, fever above 37.5 °C, reticulin myelofibrosis (MF), mild lymphadenopathy, and progressive renal insufficiency and with other diseases excluded, we diagnosed this patient as having TAFRO syndrome according to the diagnostic criteria [3]. The administration of intravenously administered tocilizumab (8 mg/kg) was begun on the same day with tapering prednisolone dose; his C-reactive protein and alkaline phosphatase levels gradually improved, along with his renal function and fever (Fig. 5). For the anasarca, furosemide and potassium canrenoate were used and were highly effective.

Because the thrombocytopenia remained, we added eltrombopag, a thrombopoietin receptor agonist, on hospital day 14, followed by tocilizumab administered on hospital day 16. Then, his platelet count began to increase. Under the strong immunosuppressive treatment, he contracted methicillin-resistant *Staphylococcus epidermidis* bacteremia on hospital day 20 and cytomegalovirus viremia on hospital day 31, which were successfully treated with vancomycin and ganciclovir, respectively. Tocilizumab was administered a third time on hospital day 47, and our patient was discharged on hospital day 48. After discharge, he remained afebrile and with an alkaline phosphate level within normal limits, and tocilizumab administration was no longer necessary. Eltrombopag administration was stopped because his platelet count increased and remained stable within normal limits. Nine months after the first treatment, this patient continues to do well. He is only being treated with low-dose prednisolone at approximately 5 mg per day and is still tapering carefully because of the presence of unilateral adrenal necrosis, considering the possibility of adrenal insufficiency.

## Discussion

Since 2010, when the first case of TAFRO syndrome was reported [1], the concept and clinicopathology of TAFRO syndrome has gradually been established [3]. However, there are only a few cases of TAFRO syndrome involving adrenal gland lesions, and there are no reports to date about TAFRO syndrome with adrenal necrosis shown pathologically. As a result, this is the first case of a middle-aged man diagnosed as having TAFRO syndrome with unilateral adrenal necrosis. The pathological investigations and the clinical course in this case are useful for understanding the etiology of TAFRO syndrome.

First, we need to discuss why this unusual unilateral adrenal necrosis occurred in this relatively rare disease. In general, the cause of adrenal necrosis ranges widely and includes hemorrhage, infarction, hemorrhagic infarction, bacterial infection, malignancy, and autoimmune inflammation, among other causes. In this patient, no contrast was observed in the left adrenal gland. The blood and adrenal tissue culture results showed no bacterial growth, and the adrenal gland biopsy showed no malignant cells or hemosiderin accumulation. These facts reduce the possibility of hemorrhagic involvement. In addition, malignant cells were not detected by the adrenal gland, bone marrow, or lymph node biopsy. Thus, we assumed that infarction or an autoimmune process was the leading cause of adrenal necrosis in this case. The possibility of infarction seemed to be higher than that of autoimmune destruction in this case. Although the imaging studies and pathological tests could not detect a thrombus itself, the fact that the adrenal lesion was unilateral increases the possibility of infarction. Moreover, adrenal lesions in autoimmune adrenalitis, or Addison’s disease, are typically bilateral, and to the best of our knowledge, no cases of unilateral adrenal lesions caused by an autoimmune process have been reported. In general, most cases of adrenal infarction are accompanied by hemorrhage and bilateral lesions versus unilateral lesions [6]. In some cases, the infarction has been suggested as a sign of antiphospholipid syndrome, essential thrombocytosis, or polycythemia vera [7–9]. There have also been several cases of unilateral adrenal infarction occurring along with pregnancy [6, 10]. Considering these findings, coagulopathy seems to be related to unilateral adrenal infarction.

Notably, prednisolone and tocilizumab therapy were highly effective in this case, which suggests that the pathophysiology of TAFRO syndrome may be relevant to autoimmune or autoinflammation processes, or hypercytokinemia. The pathophysiology of TAFRO syndrome is not yet fully understood. TAFRO syndrome is thought to be classified as iMCD [2], but the etiology of iMCD itself also remains unknown. Recently, the Castleman Disease Collaborative Network proposed a hypothesis of the etiological factors of iMCD, including autoimmunity, autoinflammation by germline mutation, neoplasm, and infection by non-HHV-8 virus [2, 11]. The involvement of autoimmunity is considered to be mediated by innate antibodies, which cause the release of several cytokines, such as interleukin-6 (IL-6) and vascular endothelial growth factor (VEGF), thereby playing an important role in clinical flares of iMCD [12]. However, there is little evidence of an association between the IL-6 level and the efficacy of anti-IL-6 antibody treatment in iMCD and TAFRO cases, suggesting the existence of other primary drivers [2, 12]. In this case, the level of IL-6 gradually decreased after the treatment, but the VEGF level did not show this trend (Fig. 5). Approximately 30% of iMCD cases, including cases with TAFRO features, exhibit autoantibody involvement, such as anti-nuclear antibodies, anti-Sjögren syndrome-A (SS-A) antibodies, or a history of autoimmune hemolytic anemia [13, 14]. In addition, in several other cases, as in this case, increased peripheral thrombocyte consumption refractory to platelet transfusion has been reported [2]. These features indicate the clinicopathological involvement of autoimmunity.

The problem is that whether autoimmunity and hypercytokinemia are the primary causes of TAFRO syndrome or secondary reactions due to triggers, such as autoinflammation, malignancy, or some viral infection, remains to be determined; thus, further research is necessary.

Therefore, we concluded that systemic inflammation caused a hypercoagulable state in this case, leading to unilateral adrenal gland infarction or severe inflammation, causing left adrenal gland enlargement and, in turn, leading to ischemia itself. In terms of the association between adrenal lesions and TAFRO syndrome, there have been a few cases of TAFRO syndrome with adrenal hemorrhage [4, 5]. Bilateral hemorrhage occurred in one case and a unilateral lesion in another. In each case, the proposed etiology for the hemorrhage was retroperitoneal inflammation, thrombocytopenia, and organomegaly of the adrenal gland itself. These were cases of adrenal hemorrhage without necrosis; there have been no previous reports of a case of TAFRO syndrome with adrenal necrosis. This is the first case of TAFRO syndrome accompanied by unilateral adrenal necrosis without evidence of hemorrhage shown by biopsy. In this case, hypercytokinemia led to ischemia of the adrenal gland, suggesting the possibility of hypercoagulability in TAFRO cases or the involvement of ischemia in organomegaly, which is one of the features of TAFRO syndrome. As the whole etiology is still unknown, further investigation of the pathophysiology of TAFRO syndrome and its influence on adrenal glands is warranted.

## Conclusions

This is the first case of TAFRO syndrome complicated with unilateral adrenal necrosis without evidence of hemorrhage. Based on the results of pathological investigation, we conclude that the unilateral adrenal necrosis in this case was caused by ischemia of the adrenal gland itself. Hypercytokinemia, which is one of the features of TAFRO syndrome, may be related to this ischemia, and immunosuppressive therapy is key for treating TAFRO syndrome.

## Abbreviations

bpm: Beats per minute
CT: Computed tomography
HHV-8: Human herpesvirus 8
IL-6: Interleukin-6
iMCD: Idiopathic multicentric Castleman disease
MF: Myelofibrosis
SS-A: Sjögren syndrome-A
VEGF: Vascular endothelial growth factor
